# Finite element analysis and biomechanical study of “sandwich” fixation in the treatment of elderly proximal humerus fractures

**DOI:** 10.3389/fbioe.2024.1425643

**Published:** 2024-07-08

**Authors:** Yang Lv, Ziyan Zhang, Ji Qv, Qian Sheng, Jian Zhang, Chengdong Piao

**Affiliations:** ^1^ Department of Orthopedics, The Second Hospital of Jilin University, Changchun, China; ^2^ Department of Radiology department, The Second Hospital of Jilin University, Changchun, China

**Keywords:** proximal humerus fracture, intramedullary support, screw extraction, finite element analysis, biomechanical experiment

## Abstract

Proximal humerus fractures (PHFs) are common in the elderly and usually involve defects in the medial column.The current standard for medial column reconstruction is a lateral locking plate (LLP) in combination with either an intramedullary fibula support or an autogenous fibula graft. However, autogenous fibula graft can lead to additional trauma for patients and allogeneic fibular graft can increase patients’ economic burden and pose risks of infection and disease transmission. The primary objective of this study was to introduce and assess a novel “Sandwich” fixation technique and compare its biomechanical properties to the traditional fixation methods for PHFs. In this study, we established finite element models of two different internal fixation methods: LLP-intramedullary reconstruction plate with bone cement (LLP-IRPBC) and LLP-intramedullary fibula segment (LLP-IFS). The biomechanical properties of the two fixation methods were evaluated by applying axial, adduction, abduction, torsional loads and screw extraction tests to the models. These FEA results were subsequently validated through a series of biomechanical experiments. Under various loading conditions such as axial, adduction, abduction, and rotation, the LLP-IRPBC group consistently demonstrated higher structural stiffness and less displacement compared to the LLP-IFS group, regardless of whether the bone was in a normal (Nor) or osteoporotic (Ost) state. Under axial, abduction and torsional loads, the maximum stress on LLPs of LLP-IRPBC group was lower than that of LLP-IFS group, while under adduction load, the maximum stress on LLPs of LLP-IRPBC group was higher than that of LLP-IFS group under Ost condition, and almost the same under Nor condition. The screw-pulling force in the LLP-IRPBC group was 1.85 times greater than that of the LLP-IFS group in Nor conditions and 1.36 times greater in Ost conditions. Importantly, the results of the biomechanical experiments closely mirrored those obtained through FEA, confirming the accuracy and reliability of FEA. The novel “Sandwich” fixation technique appears to offer stable medial support and rotational stability while significantly enhancing the strength of the fixation screws. This innovative approach represents a promising strategy for clinical treatment of PHFs.

## 1 Introduction

Proximal humerus fractures (PHFs) are the common type of injury, accounting for approximately 4%–9% of all adult fractures (Badman and Mighell, 2008). The incidence of these fractures tends to increase with age, owing to reduced bone mineral density among the elderly (Sun et al., 2020). Typically, low-energy falls, particularly in older individuals, are the primary mechanisms leading to PHFs (Bergdahl et al., 2016). Nondisplaced or minimally displaced PHFs are generally managed conservatively, while displaced and unstable PHFs require open reduction and internal fixation (Laux et al., 2017). Among the surgical options for displaced PHFs, locking plates are the most commonly employed (Bergmann et al., 2011). However, these locking plates are typically positioned on the lateral aspect of the humerus, often neglecting the mechanical stability of the medial column. In cases where the medial bone is comminuted or osteoporotic (Ost), postoperative complications such as varus collapse, screw cutout, internal fixation failure, and osteonecrosis can occur at rates as high as 39% (Robinson et al., 2003; Boesmueller et al., 2016).

Numerous studies have established that effective reconstruction of the medial column can significantly reduce the incidence of PHF complications (Zhu et al., 2014; Chen et al., 2015; He et al., 2015; Jabran et al., 2018). The prevailing method for medial column reconstruction in clinical practice involves the use of allogeneic or autologous fibular strut marrow support (Chen et al., 2018; Cui et al., 2019; Lee et al., 2019; Wang et al., 2019). However, the availability of allogeneic fibula is somewhat limited, which places an increased financial burden on patients. Additionally, the use of autogenous fibula segments can lead to additional trauma for patients (Mease et al., 2021). Therefore, this study proposes a “Sandwich” method for treating PHFs, which involves the application of double plates (a lateral locking plate and a medial medullary plate) in combination with bone cement.

The primary objective of this study was to compare the biomechanical characteristics of “Sandwich” fixation with the traditional fixation of using a locking plate combined with intramedullary fibular segment transplantation, and to evaluate the practicability of “sandwich” fixation in the reconstruction of the medial column of PHFs by finite element analysis (FEA) and biomechanical experiments. Ultimately, this research endeavors to provide a theoretical foundation for the selection of appropriate internal fixation methods in clinical practice.

## 2 Methods

Computed tomography scans of the right humerus was selected from a healthy 30-year-old female volunteer with no history of injury or pathological disease, such as scapulohumeral periarthritis, bone disease, or bone tumor. We established finite element models of LLP-IRPBC and LLP-IFS, and applied axial, adduction, abduction, torsional loads and screw extraction tests to the models to evaluate the biomechanical properties of the two fixation methods. Subsequently, 20 right prosthetic humeri were randomly divided into 2 groups for *in vitro* biomechanical experiments to verify the finite element analysis results. The research was approved by the ethics committee of The Second Hospital of Jilin University (No. 2023-211), and all procedures were conducted in accordance with relevant guidelines and regulations. Written informed consent was obtained from the volunteer prior to participation in the study.

### 2.1 Finite element modeling

Computed tomography scans of the right humerus in DICOM format were imported into Mimics 21.0 software (Materialise, Leuven, Belgium) to create a 3D model of the humerus. The Hounsfield Unit (HU) value, representing the bone density threshold value, was utilized to differentiate cortical and cancellous bone. Cancellous bone was defined by an HU value of 150–450, while cortical bone was defined by an HU value of 450–3000 (Chen et al., 2020). The model was then exported in stereolithography format to Geomagic Warp 2014 (3D Systems, North Carolina, United States). Nails and redundant features of the model were removed, and the model was smoothed. Accurate surface modules were used to identify model contours, edit any distorted or unreasonable contours, and add additional contours to facilitate surface patch generation. After successful surface patch generation, the surface was fitted and exported in STEP format.

**FIGURE 1:**
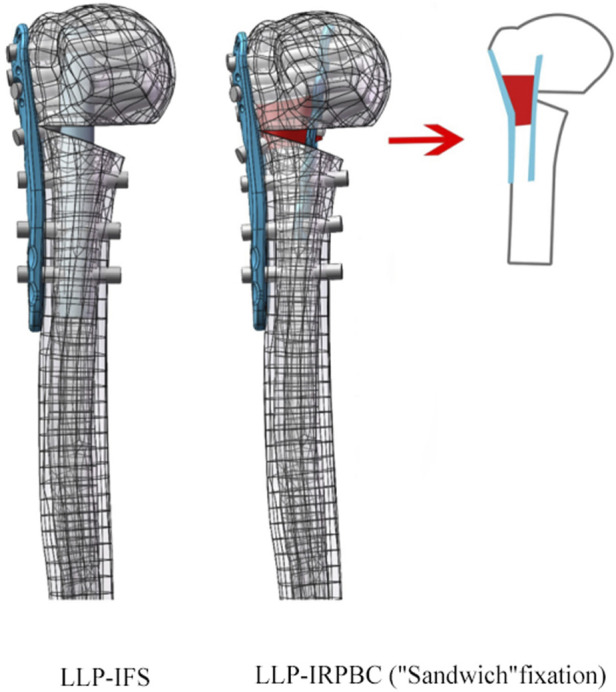
Two-part fracture model of proximal humerus with an unstable medial column. Two types of fixation configurations, LLP-IFS = lateral locking plate-intramedullary fibula segment; LLP-IRPBC (“Sandwich” fixation) = lateral locking plate-intramedullary reconstruction plate with bone cement.

To generate STP-format models, 1:1 scans of the lateral locking plate (LLP, 90 mm in length, 22 mm in width, Zimmer, Indiana, United States) and the reconstructed plate (65 mm in length, 10 mm in width, Zimmer, Indiana, United States) were conducted in advance. These models were then assembled with the humerus model using Solidworks 2017 (Dassault Systèmes, Massachusetts, United States). The model was standardized by performing medial wedge osteotomy, according to previous studies, creating a 10-mm fracture gap with complete lateral contact (Zettl et al., 2011). Implants were placed according to the manufacturer’s guidelines. In the LLP-IFS (lateral locking plate-intramedullary fibula segment) group, the LLP and fibular strut were implanted into the model, with a hollow cylinder used to simulate the fibular strut. The hollow cylinder had a length of 85 mm, an outer radius of 5 mm, and an inner radius of 2 mm (He et al., 2017). In the LLP-intramedullary reconstruction plate with bone cement (LLP-IRPBC) group, the reconstruction plate and LLP were inserted into the model, and a short screw was placed into the third nail hole of the reconstruction plate. The 7th and 8th screws on the LLP passed through the reconstruction plate simultaneously. A solid cylinder, representing bone cement (27 mm in length, 7 mm radius, wherein the size was measured after the following conditions were met), was inserted between the LLP and the reconstruction plate through the fracture space, connecting the two plates and encasing the 5th, 6th, and 7th screws, as well as the screws on the reconstruction plate, creating a stable “Sandwich” structure (Figure 1).

This study utilized hexahedral meshing for simple components such as screws, bone cement, and fibula segments to save computation time and ensure result accuracy, while tetrahedral meshing was applied to more complex structures such as the humerus, locking plates, reconstructed plates, and screw thread for more accurate stress and strain calculations. The sensitivity analysis conducted in this study focused on mesh density, confirming that mesh density does not significantly impact the calculated results. After meshing and sensitivity analysis, the LLP-IFS model consisted of 238842 elements and 383894 nodes, while the LLP-IRPBC model comprised 245355 elements and 392323 nodes.

### 2.2 Finite element analysis

**FIGURE 2:**
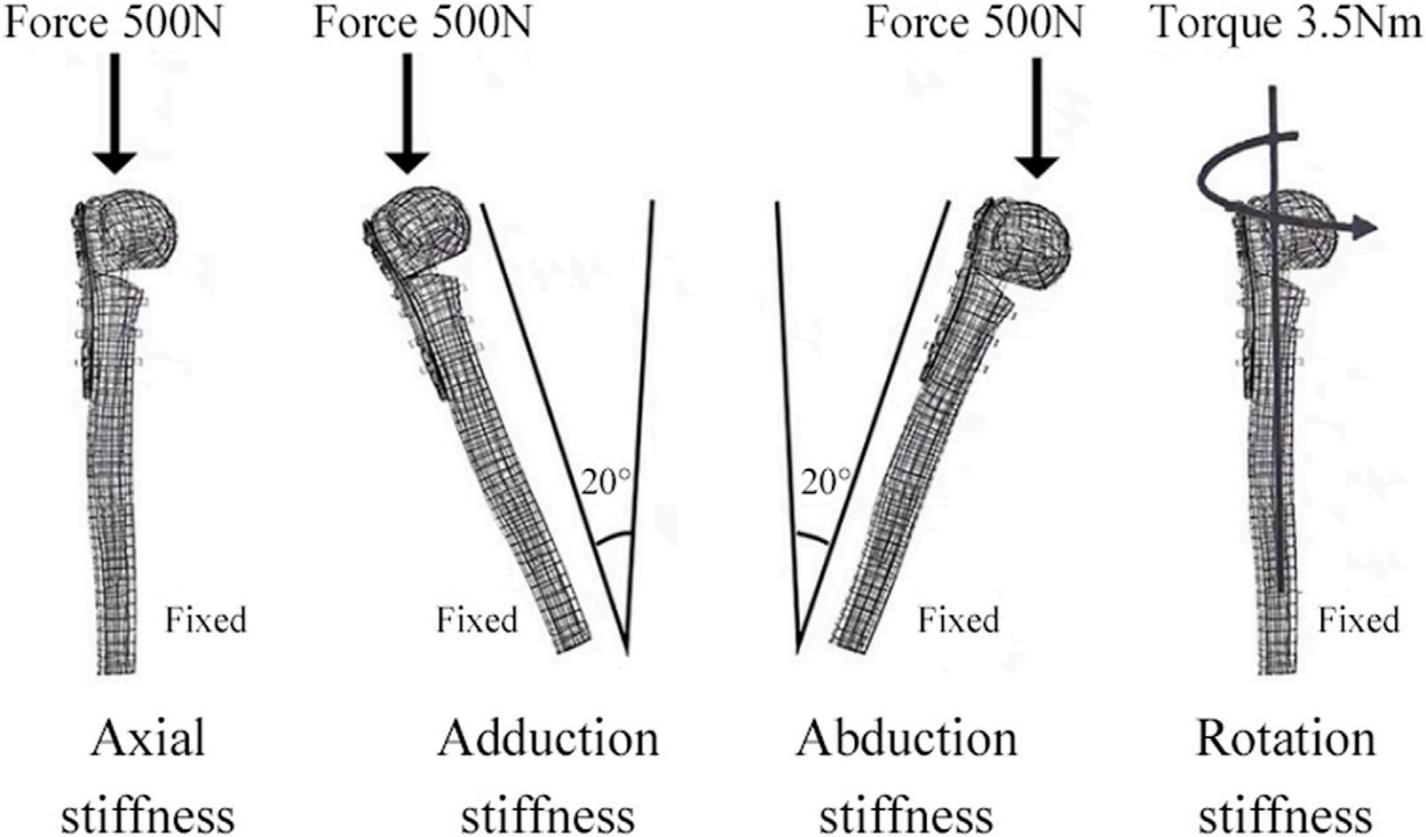
The distal shaft of the humerus was fixed. Axial, adductive, abductive, and torsional forces were applied to the head of the humerus.

**FIGURE 3:**
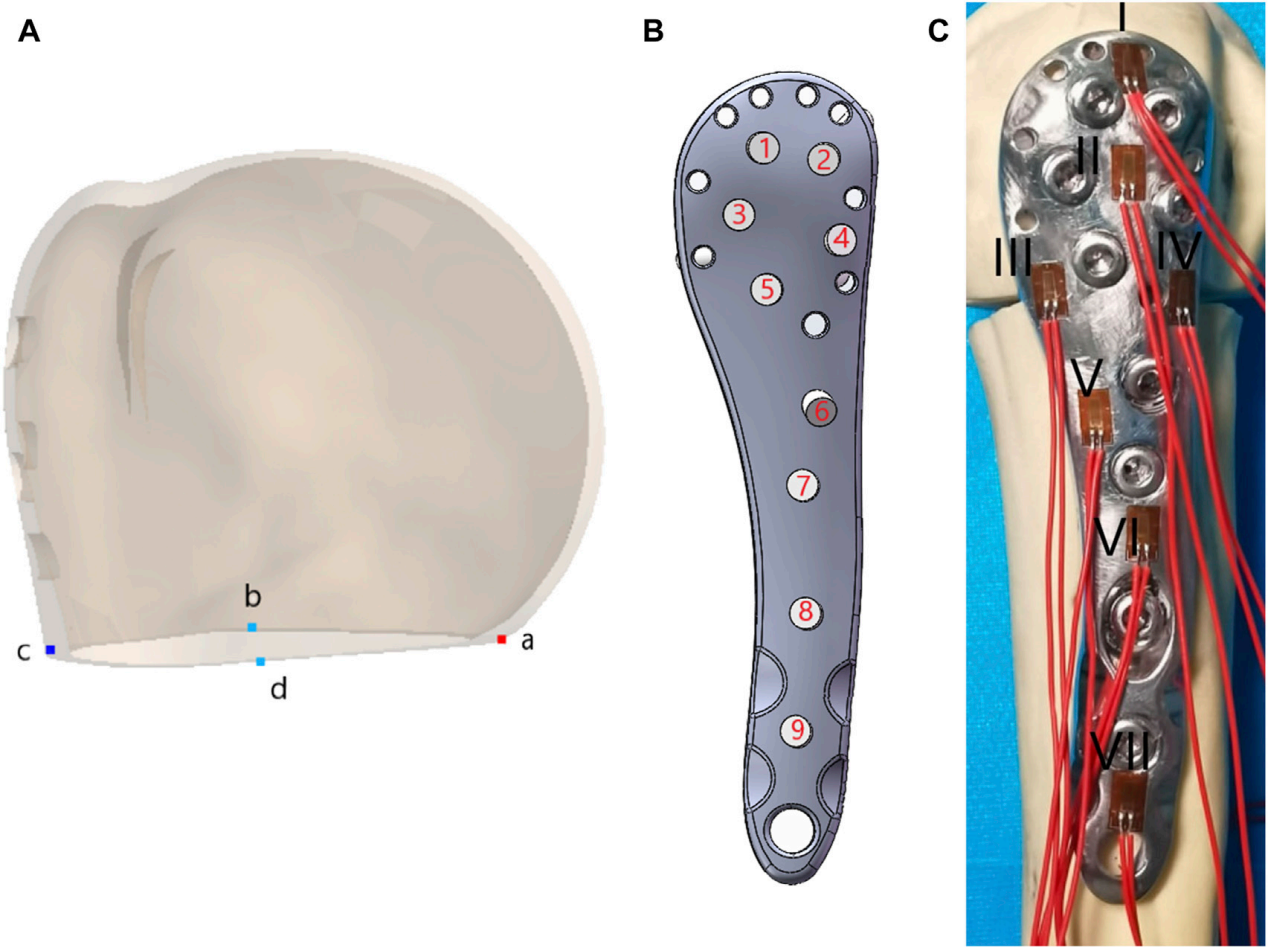
**(A)** Four points on the proximal fracture gap medial (a), anterior (b), lateral (c), and posterior (d); **(B)** Screw numbers on the lateral locking plate; **(C)**Position distribution and numbering of the strain gauge.

FEA was conducted using Ansys Workbench 17.0 (ANSYS, Inc., Pittsburgh, United States). All models and implants were assumed to be isotropic, homogeneous, and linear elastic, and they simulated bone stock in both normal (Nor) bone and Ost bone. The elastic modulus for cortical bone, Ost cortical bone, cancellous bone, Ost cancellous bone, and implant were set at 13400 MPa, 2000 MPa, 8844 MPa, 660 MPa, and 114000 MPa, respectively, with a Poisson’s ratio of 0.3 (He et al., 2015). Bone cement had an elastic modulus of 2270 MPa and a Poisson’s ratio of 0.46 (Zheng et al., 2020). Contact behavior between plate/locking screws, locking screws/bone, and locking screws/bone cement interface was defined as bonded. All contact elements were defined as deformable elements. The distal end of the PHF model was mechanically constrained with 6 degrees of freedom. Axial, adduction, abduction, and torsion forces were applied to the model (Figure 2). Axial loads of 500 N were applied to the proximal humeral head in both coronal and sagittal planes, vertically oriented. To simulate adduction and abduction forces, the model’s angle was adjusted by 20° in either adduction or abduction based on axial circumstances. To simulate rotation, a 3.5-Nm torque was applied around the humeral head at the proximal end of the humeral axis. Fixation device stability was evaluated by observing displacement and angle changes of the osteotomy gap under axial, adduction, abduction, and rotational loads. Four points on the proximal fracture gap—medial (a), anterior (b), lateral (c), and posterior (d)—were selected for measuring displacement (Figure 3A). To evaluate stress distribution and force conditions, the von Mises stress distribution and maximum stress on the implant were determined.

### 2.3 Screws extraction experiments

In this study, we focused on screw No. 7 (screw numbers are indicated in Figure 3B) to conduct a nail extraction experiment assessing screw stability with different fixation methods. We simplified the model and added threads to the screws with a screw length of 25 mm and a pitch of 1.75 mm. All contact types were set according to Coulomb’s friction law, with friction coefficients of 0.3 for screw/bone and screw/bone cement interactions (Kayabasi and Ekici, 2007). The maximum strengths of Nor cortical bone, Nor cancellous bone, Ost cortical bone, Ost cancellous bone, and bone cement were set at 150 MPa, 13 MPa, 85 MPa, 10 MPa, and 33.6 MPa, respectively (McCalden et al., 1993; Anglin et al., 1999; Hart et al., 2017; Khellafi et al., 2019). The maximum strength of the screw was 3000 MPa, as determined by a tensile test (*f = F/A*). The outer surface of the bone material was constrained in translational degrees of freedom in three directions. Contact was established at the screw connection, between the screw, the bone, and the bone cement. After meshing and sensitivity analysis, both sets of pull-out models consisted of 217,700 elements and 438,090 nodes. The load was applied to the screw head, and large deformation settings were enabled during the calculations to determine the pull-out force required for screw extraction.

### 2.4 Experimental specimens and preparation

Synbone prosthetic humeri (No. 5010, SYNBONE-AG, Switzerland) were employed in this study. A total of 20 right prosthetic humeri were randomly allocated into two groups, each consisting of 10 prosthetic humeri: the LLP-IFS group and the LLP-IRPBC group. Subsequently, an experienced surgeon performed a transverse osteotomy using an oscillating saw. The osteotomy procedure was consistent with the FEA methods described above. The placement of implants followed the manufacturer’s guidelines. The locking plate was positioned 8 mm distal to the greater tuberosity, and 3.5-mm screws were inserted until they were 5–8 mm from the joint surface, guided by fluoroscopy. In the LLP-IFS model, LLP and a fibular strut were implanted, creating the LLP-IFS model. In the LLP-IRPBC group, a custom-molded reconstruction plate was inserted into the medullary cavity after placing a short screw into the third nail hole. The fracture was reduced, and then the locking plate and screws were positioned according to the manufacturer’s guidelines. The 7th and 8th screws on the LLP plate were passed through the reconstruction plate simultaneously using fluoroscopy. Bone fenestration was conducted above and below the fracture line, and bone cement (PALACOS R + G, Heraeus Medical GmbH, Germany) was placed between the two plates. This allowed the bone cement to connect the two plates and encircle the 5th, 6th, and 7th screws as well as the screws on the reconstruction plate concurrently. Finally, the loose bone fragments were restored.

The distal part of the humerus was then sectioned approximately 22 cm from the humeral head apex, and the distal end was securely fixed using dental plaster (Type-II, self-curing, Xinshiji, Shanghai, China). To measure the maximum stress on the LLP under three loading modes—axial, adduction, and abduction—we applied strain gauges (120-2AA-D-D300, Guangce Electronics Co., Ltd., Hunan, China) to the steel plate’s surface. The distribution of strain gauge positions is depicted in Figure 3C. The strain gauge leads were connected to a strainmeter by wires and cables, with external temperature compensation.

### 2.5 Biomechanical testing

**FIGURE 4:**
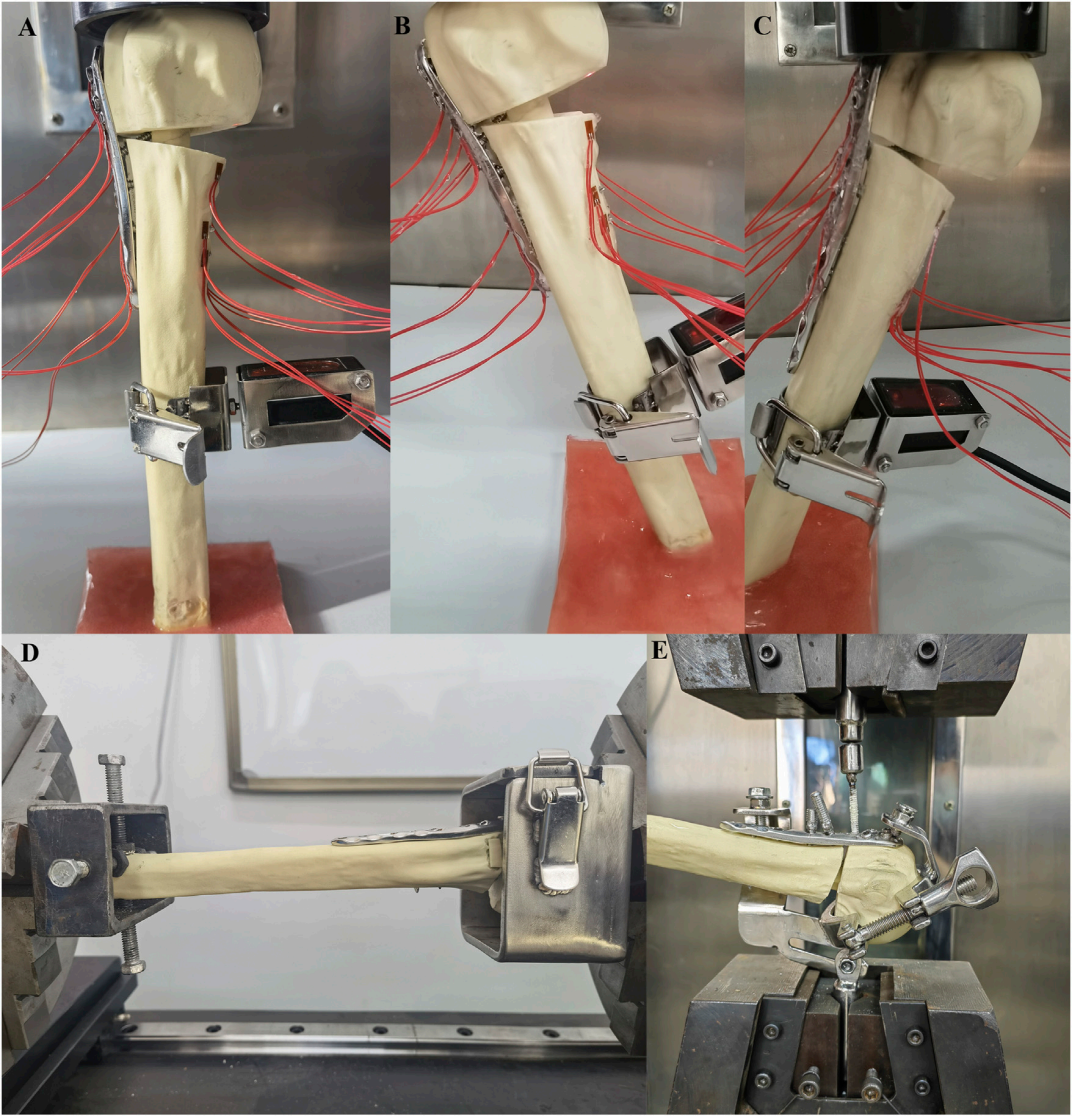
The distal part of the specimens were fixed and placed in three positions (0° axial, −20° adduction, +20° abduction), and the compression load was applied to the humerus head at a speed of 2 mm/min **(A–C)**; The humerus head and distal ends were fixed with blunt screws and connected to the torsion tester using custom clamps, and the torque forces were applied to the humeral head at 0.1°/s until the torque reached 3.5 Nm **(D)**; The humerus was fixed, and the tensile force was applied vertically to the nut at a speed of 2 mm/min until the screw was completely removed **(E)**.

All compression tests were conducted using an MTS model 55,100 material testing machine (Material Testing Systems, MTS Systems Corp, Eden Prairie, MN, United States; Figure 4). The distal ends of the specimens were fixed and oriented vertically in the coronal and sagittal planes, with the specimens sequentially positioned at three angles (0° axial, −20° adduction, and + 20° abduction). The compression load was applied to the humerus head at a speed of 2 mm/min using a load sensor, with each specimen undergoing five cycles between 50 N and 500 N. Relative displacement between the broken fragments was measured using a laser displacement transducer (HG-C1100, range 35 mm, resolution 0.07 mm, Lingguang Technology, Anhui, China), and axial stiffness was calculated based on the slope of the linear region of the load-displacement curve. The strain value at each measuring point under a 500 N load was measured using a strainmeter, and stress values were calculated based on δ = ε × Ε. Before testing, the specimens were preloaded with 100 N to eliminate rheological effects such as bone relaxation and creep (Kim et al., 2001).

In torsional tests, the humerus head and distal ends were secured with blunt screws and connected to the torsion tester (NWS-1000, China Machinery Testing Equipment Co., Ltd., Changchun, China) using custom clamps. Torque forces were applied to the humeral head at a rate of 0.1°/s until a torque of 3.5 Nm was reached. Each specimen underwent five cycles, with torsional stiffness calculated based on the slope of the linear region of the torsion-torsion angle curve.

For the screw extraction experiments, we used the MTS model 55100 material testing machine, with the humerus and screw respectively fixed onto a custom mold with adjustable angles. The humerus was immobilized, and tensile force was applied vertically to the nut at a rate of 2 mm/min until the screw was completely removed. The maximum force required for complete extraction of screws No. 5, 6, and 7 (through the fibula in the LLP-IFS group and the cement in the LLP-IRPBC group) was recorded for the two groups of specimens.

### 2.6 Statistical analysis

All data were subjected to statistical analysis using SPSS 20.0 (SPSS Inc., Chicago, IL, United States) software. The Kolmogorov-Smirnov test was employed to assess data normality, and a *t*-test was used to compare data between the two groups. A significance level of *p* < 0.05 was considered statistically significant.

## 3 Results

### 3.1 Construct stiffness

**FIGURE 5:**
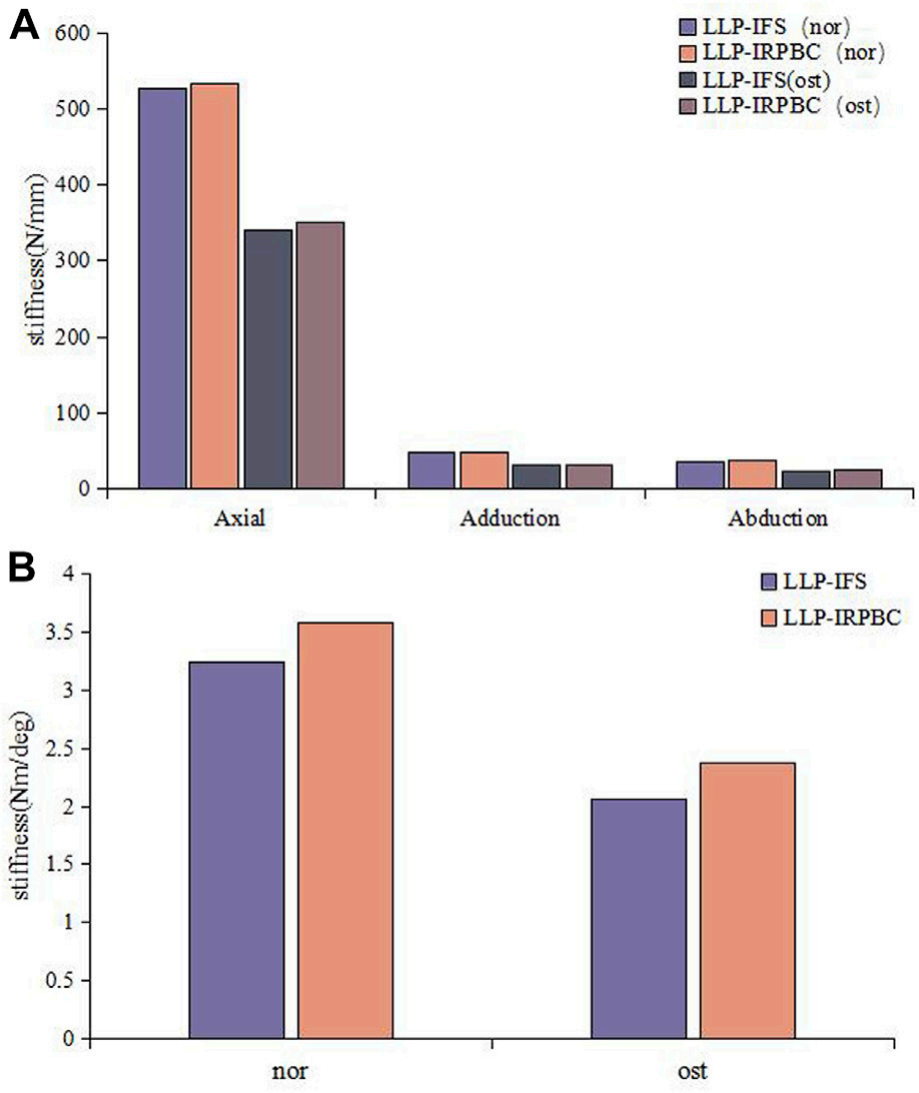
Results of structural stiffness. **(A)** Axial force, adduction force, abduction force; **(B)** Rotational force.

Axial, adduction, abduction, and torsion forces were applied to the LLP-IFS group and LLP-IRPBC group under both Nor and Ost conditions, the structural stiffness of LLP-IFS group under Nor conditions was 527.50N/mm, 47.81N/mm, 36.65N/mm and 3.25Nm/deg, while that of LLP-IRPBC group was 533.62N/mm, 48.12N/mm, 38.50N/mm and 3.58Nm/deg, and the structural stiffness of LLP-IFS group under Ost conditions was 340.76N/mm, 31.12N/mm, 23.83N/mm and 2.06Nm/deg, while that of LLP-IRPBC group was 350.30N/mm, 31.54N/mm, 25.23N/mm, and 2.37Nm/deg. For the four loads, the LLP-IRPBC group exhibited higher structural stiffness compared to the LLP-IFS group under both Nor and Ost conditions, as shown in Figures 5A,B.

### 3.2 Relative fracture displacement

**FIGURE 6:**
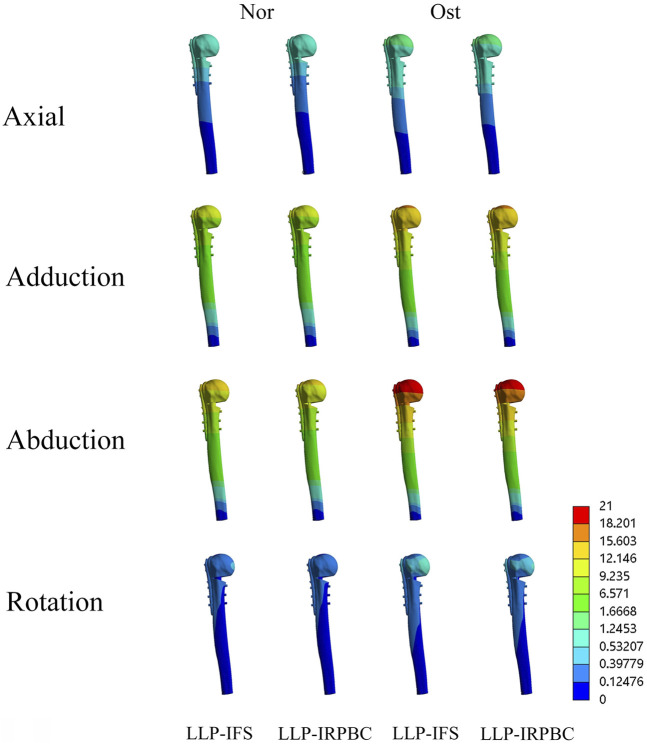
The displacement cloud diagram.

**FIGURE 7:**
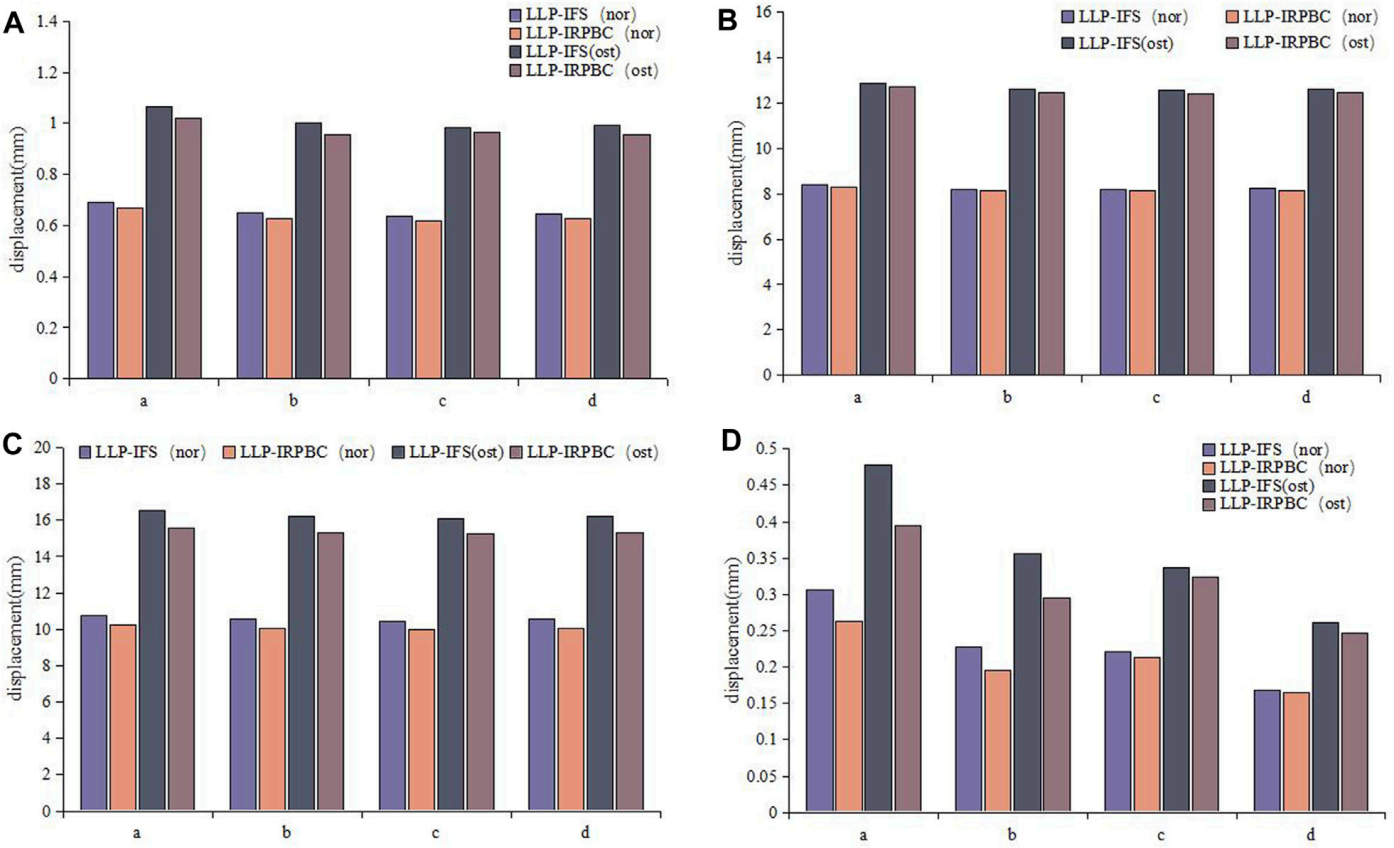
Relative displacement of fracture gap. **(A)** Axial load; **(B)** Adduction load; **(C)** Abduction load; **(D)** Rotation load.

Figures 6, 7 illustrates the displacement cloud diagram and the results of amplitude of distance.

Under axial load, the displacements of a, b, c and d of LLP-IFS group (Nor/Ost) were 0.69 mm/1.06 mm, 0.65 mm/1.00 mm, 0.64 mm/0.98 mm, 0.64 mm/0.99 mm, while those of LLP-IRPBC group were 0.67 mm/1.02 mm, 0.63 mm/0.96 mm, 0.62 mm/0.96 mm and 0.63 mm/0.96 mm, respectively, as depicted in Figure 7A.

Under adduction load, the displacements of a, b, c and d of LLP-IFS group (Nor/Ost) were 8.37 mm/12.86 mm, 8.21 mm/12.61 mm, 8.17 mm/12.55 mm, 8.24 mm/12.62 mm, while those of LLP-IRPBC group were 8.31 mm/12.69 mm, 8.15 mm/12.45 mm, 8.12 mm/12.38 mm and 8.15 mm/12.45 mm, respectively, as portrayed in Figure 7B.

Under abduction load, the displacements of a, b, c and d of LLP-IFS group (Nor/Ost) were 10.78 mm/16.57 mm, 10.53 mm/16.20 mm, 10.46 mm/16.09 mm, 10.57 mm/16.19 mm, while those of LLP-IRPBC group were 10.27 mm/15.56 mm, 10.04 mm/15.32 mm, 9.98 mm/15.23 mm and 10.03 mm/15.31 mm, respectively, as illustrated in Figure 7C.

Under rotational load, the displacements of a, b, c and d of LLP-IFS group (Nor/Ost) were 0.31 mm/0.48 mm, 0.23 mm/0.36 mm, 0.22 mm/0.34 mm, 0.17 mm/0.26 mm, while those of LLP-IRPBC group were 0.26 mm/0.39 mm, 0.19 mm/0.30 mm, 0.21 mm/0.32 mm, and 0.16 mm/0.25 mm, respectively, as evidenced in Figure 7D.

Under the above loading conditions, regardless of whether it was in Ost or Nor conditions, the displacements at points a, b, c, and d in the LLP-IRPBC group were consistently smaller than those in the LLP-IFS group, and the fracture amplitude of distance were the largest under abductive loads.

### 3.3 Von Mises stress distribution and maximum stress on implants

**FIGURE 8:**
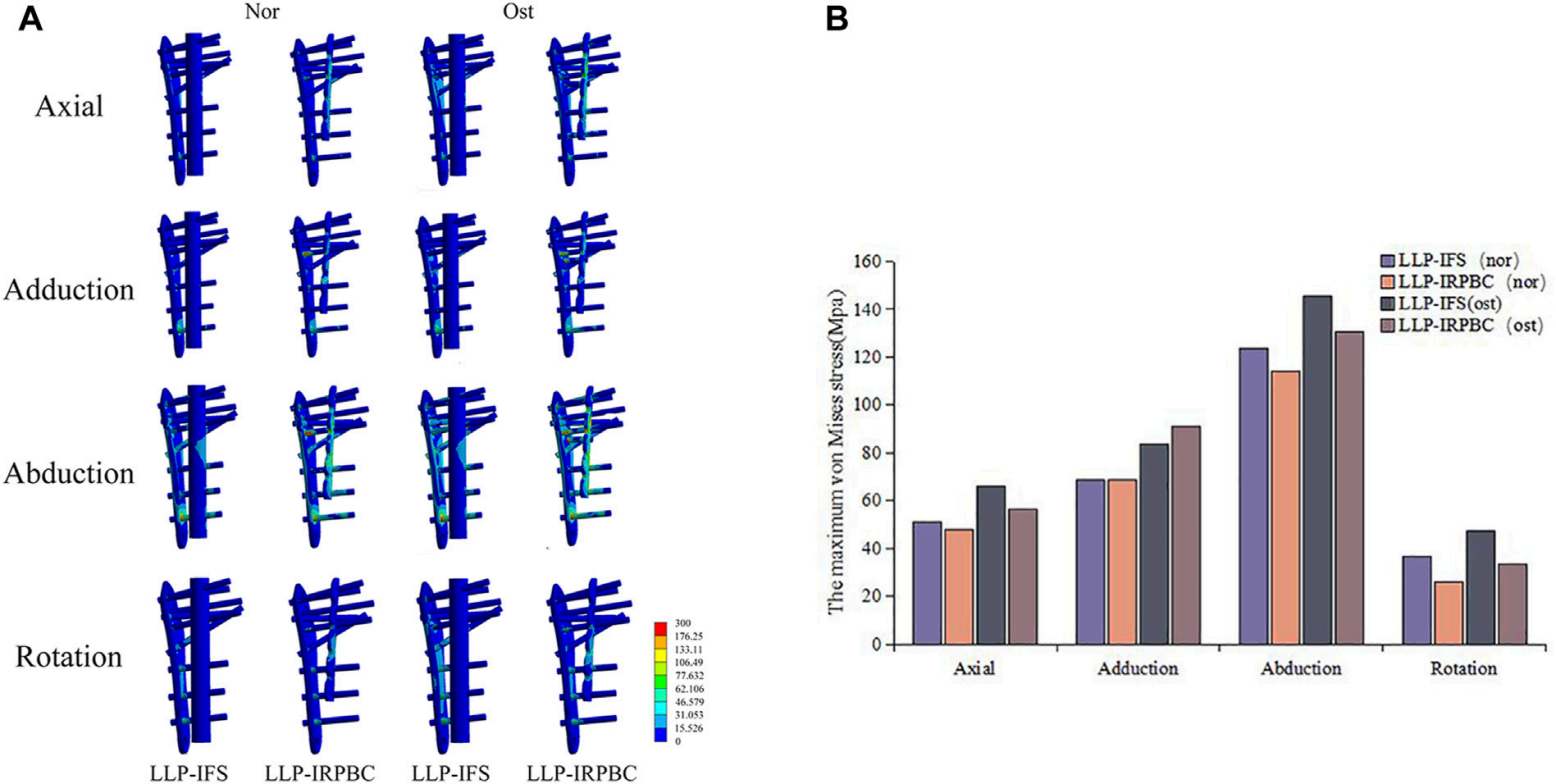
**(A)** Von Mises stress distribution of the lateral locking plate (LLP)-intramedullary fibula segment (IFS) and LLP-intramedullary reconstruction plate with bone cement (IRPBC) under Nor and Ost conditions. **(B)**. Maximum von Mises stress on the lateral locking plates.

The von Mises stress distribution and maximum stress of the implant are detailed in Figures 8A,B. Under axial, adduction, abduction, and torsional loads, the maximum stresses on LLPs in the LLP-IFS group (Nor/Ost) were 50.94Mpa/66.32 Mpa, 68.56 Mpa/83.61 Mpa, 123.50 Mpa/145.44 Mpa, 36.51 Mpa/47.31 Mpa, while those of LLP-IRPBC group (Nor/Ost) were 48.12 Mpa/56.54 Mpa, 68.53 Mpa/91.39 Mpa, 114.16 Mpa/130.41 Mpa, 25.96 Mpa/33.42 Mpa.

Both the LLP-IRPBC and LLP-IFS groups effectively dispersed stress through the additional implants/grafts of the locking plate. The von Mises stress distribution illustrates that both methods follow a double-column conduction mechanical pathway. In the LLP-IRPBC group, stresses concentrated around the cemented column-screw junction, primarily dispersed by intramedullary implants, with a notable stress concentration at the screw-implant junction. Von Mises stress remained similar under both Nor and Ost conditions.

### 3.4 Screws extraction experiments

**FIGURE 9:**
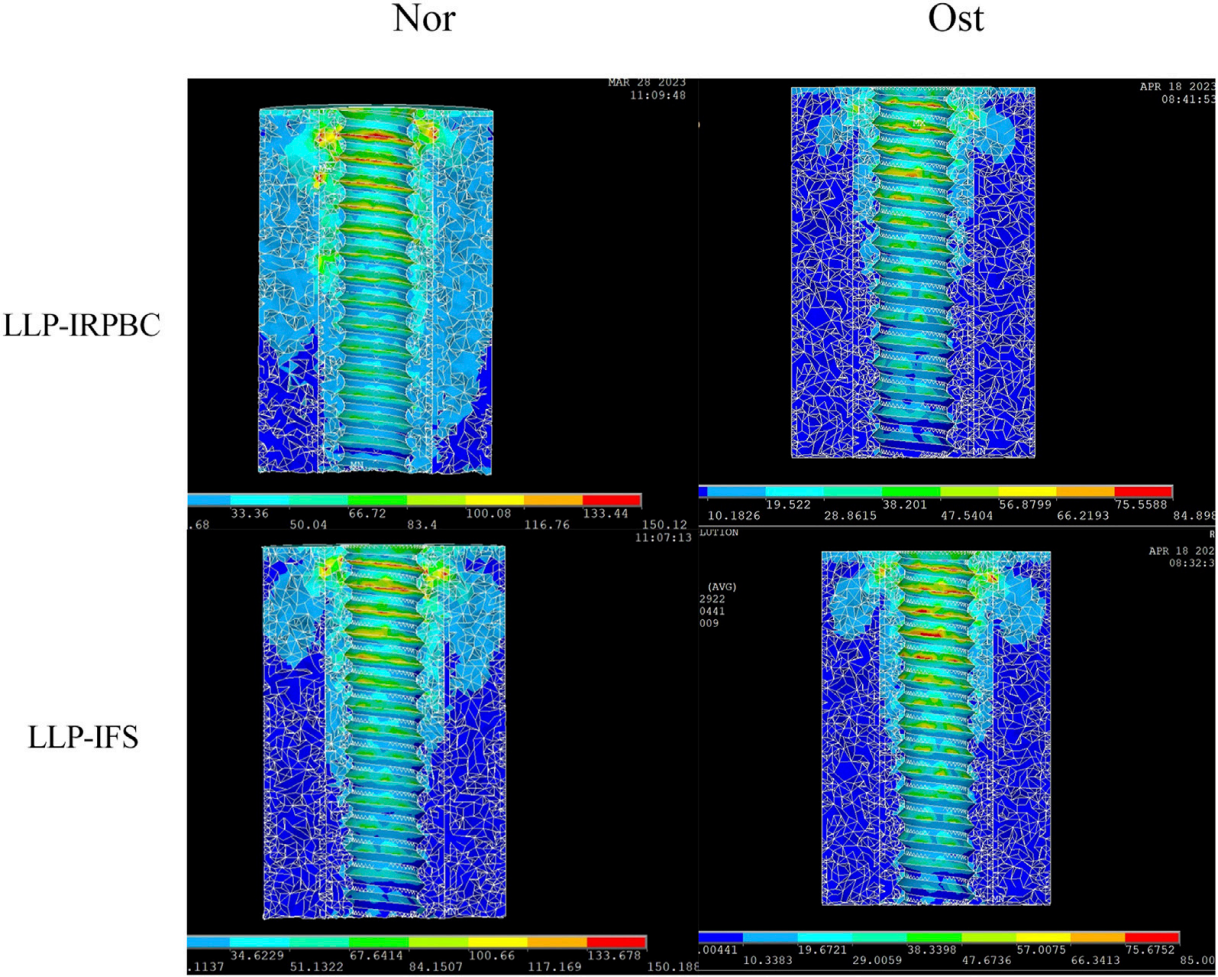
Maximum von Mises stress distribution during screw extraction.

Under Nor conditions, the force required for complete screw pull-out was 7700 N in the LLP-IRPBC group and 4150 N in the LLP-IFS group. The extraction force in the LLP-IRPBC group was approximately 1.85 times higher than that in the LLP-IFS group. Under Ost conditions, the pull-out force for the LLP-IRPBC group and LLP-IFS group were 3750 N and 2750 N, respectively. The LLP-IRPBC Ost group’s pull-out force was 1.36 times that of the LLP-IFS group. Stress distribution results and details are presented in Figure 9 and Table 1.

**TABLE 1:** The screw pull-out force of two groups.

	Screw pull-out force under Nor conditions (N)	Screw pull-out force under Ost conditions (N)
LLP-IRPBC	7700	3750
LLP-IFS	4150	2750

### 3.5 Biomechanical results

**FIGURE 10:**
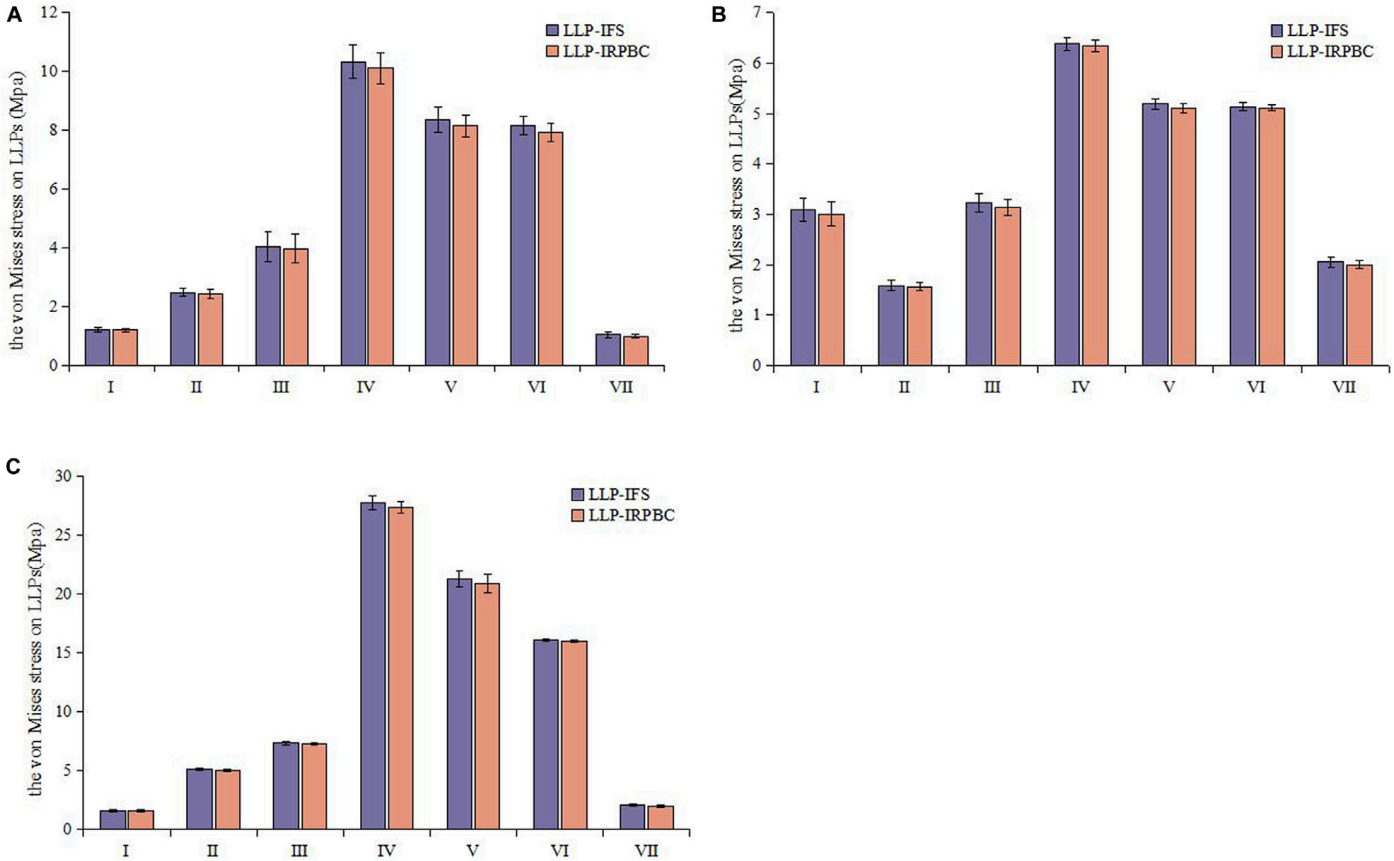
Von Mises stress distribution of the lateral locking plate. **(A)** Axial load; **(B)** Adduction load; **(C)** Abduction load.

Biomechanical tests corroborated the structural stiffness results observed in FEA. The structural stiffness of the LLP-IRPBC group was significantly higher than that of the LLP-IFS group under axial, abduction, and torsional loads (Table 2). However, there was no statistically significant difference between the two groups under adduction loads. Maximum stress on the LLPs was consistently observed at position IV for both groups under all three loading modes, with the LLP-IRPBC group showing lower stress values, although the difference was not statistically significant (*p* > 0.05; Figure 10).

**TABLE 2:** Mean stiffness with standard deviations of all testing modes.

	Axial stiffness (N/mm)	Adduction stiffness (N/mm)	Abduction stiffness (N/mm)	Rotation stiffness (Nm/deg)
LLP-IRPBC	595.7610 ± 8.22823	50.9680 ± 2.74114	42.9000 ± 2.25694	3.1630 ± 0.19726
LLP-IFS	586.1110 ± 8.17089	51.2190 ± 2.81655	40.4410 ± 1.71698	2.8680 ± 0.21872
*p*-value	0.017*	0.842	0.014*	0.005**

^*^ p< 0.05,**p< 0.01.

To prevent damage to the customized mold, based on the aforementioned FEA results, the LLP-IRPBC group ceased loading the screw when the extraction force exceeded 9900 N. Consequently, as displayed in Table 3, the pull-out force for screw No. 5 in the LLP-IRPBC group was over 2.16 times that of the LLP-IFS group, while screw No. 6 exceeded 1.64 times, and screw No. 7 was more than 2.45 times higher.

**TABLE 3:** The mean screw pull-out force with standard deviations of two groups.

	Pull-out force for screw No. 5 (N)	Pull-out force for screw No. 6 (N)	Pull-out force for screw No. 7 (N)
LLP-IRPBC	>9900	>9900	>9900
LLP-IFS	4578.8 ± 89.26598	6023.6 ± 114.46416	4042.5 ± 97.61745

### 3.6 Comparison of biomechanical experimental with finite element analysis results

The *in vitro* biomechanical experiment results were compared with the finite element analysis results for both groups under normal bone conditions, as shown in Tables 4, 5. When comparing the pulling force of screws, the average pulling force of screws No. 5, 6, and 7 was taken *in vitro*. The *in vitro* biomechanical experiment results and the finite element analysis results, with deviations less than 20%.

**TABLE 4:** Comparison of biomechanical experimental results with finite element analysis of LLP-IFS model.

	Axial stiffness (N/mm)	Adduction stiffness (N/mm)	Abduction stiffness (N/mm)	Rotation stiffness (N/deg)	Pull-out force(N)
FEA	527.5	47.81	36.65	3.25	4150
Biomechanics	586.11	51.22	40.44	2.87	4881.63
Difference	11.11%	7.10%	10.34%	11.69%	17.60%

**TABLE 5:** Comparison of biomechanical experimental results with finite element analysis of LLP-IRPBC model.

	Axial stiffness (N/mm)	Adduction stiffness (N/mm)	Abduction stiffness (N/mm)	Rotation stiffness (N/deg)	Pull-out force(N)
FEA	533.62	48.12	38.5	3.58	7700
Biomechanics	595.76	50.97	42.9	3.16	>9900
Difference	11.64%	5.92%	11.43%	13.31%	-

## 4 Discussion

Currently, open reduction and plate internal fixation represent the most commonly employed surgical approach for PHFs. However, this approach is still associated with a high incidence of postoperative complications such as varus deformity and screw removal (Südkamp et al., 2009; Sproul et al., 2011; Laux et al., 2017). Medial support reconstruction in the proximal humerus is crucial to reduce the occurrence of these complications. The intra- and extramedullary assembly fixation is better able to prevent the varus collapse for elderly proximal humeral fractures (Zhu et al., 2023a). Allogeneic or autologous fibular bone marrow grafting has emerged as a well-established and widely used method for medial column reconstruction (Gardner et al., 2008; Chen et al., 2015; Panchal et al., 2016). Nevertheless, the availability of autologous fibular struts is limited and may entail donor site complications, whereas allogeneic fibular struts can increase patients’ economic burden and pose risks of infection and disease transmission. Therefore, this study introduces a novel surgical fixation technique known as “Sandwich” fixation. It utilizes FEA and biomechanical experiments with artificial biomimetic bones to compare and analyze the biomechanical characteristics of “Sandwich” fixation and the traditional locking plate combined with fibular strut grafting. The aim is to explore the potential value of “Sandwich” fixation in medial column reconstruction for PHFs, thereby providing a theoretical foundation for clinical internal fixation selection.

By comparing with the results of Zhu et al. (2023b) and Chang et al. (2023), the reliability and high fidelity of the finite element model in this study are confirmed. The results from FEA and biomechanical studies indicate that the LLP-IRPBC group exhibits greater structural stiffness compared to the LLP-IFS group. Fracture displacement results closely correlate with structural stiffness. The LLP-IRPBC group displays smaller displacements at points a, b, c, and d under all loading conditions when compared to the LLP-IFS group. Both fixation methods offer direct two-column support and rotational stability, with LLP-IFS transmitting force through the fibula segment and LLP-IRPBC using a reconstructed steel plate. The latter provides more robust internal support due to the significantly higher stiffness of the plate compared to the fibula. The reconstructed plate forms a stable “sandwich” structure when tightly integrated with the locking plate using bone cement and screws, thereby enhancing LLP-IRPBC’s rotational stability. Overall, LLP-IRPBC provides superior structural stability, thereby promoting fracture healing and early recovery.

The inclusion of additional implants or reconstruction plates within the medullary cavity can effectively reduce the risk of implant failure by distributing stress. Our data reveals that, under axial, abduction, torsional loads, the maximum stress in the LLP-IRPBC group was exhibited lower than that in the LLP-IFS group under both Ost and Nor conditions, this difference may be attributed to the additional bone cement pathways in the LLP-IRPBC group. Interestingly, under adduction loads, the LLP-IRPBC group exhibited higher maximum stress than the LLP-IFS group in Ost conditions and the same maximun stress in Nor conditions, likely due to the gradual alignment of force direction with the screw orientation, effectively transferring the load and stress to the locking plate via the screws. Biomechanical experiment results are consistent with the findings from FEA, the maximum stresses on the LLPs almost occur at the bone defect sites, which are the most susceptible areas for failure after internal fixation. Notably, the stress under abduction load increases significantly, but it remains well below the material yield stress (800 MPa).

Annually, worldwide, over 1 million screw failures occur due to loose or displaced screws (Brown et al., 2013), often necessitating costly surgical interventions and subjecting patients to secondary trauma. To overcome these challenges, the use of bone cement to reinforce screws is often considered. Numerous *in vitro* biomechanical studies have demonstrated that bone cement-reinforced screws can enhance the initial stability of proximal humeral plates (Jabran et al., 2018; Biermann et al., 2019). An *in vivo* biomechanical study conducted by Larsson et al. (2012) revealed that calcium phosphate bone cement significantly increased screw extraction strength. A finite element study shows that bone cement enhanced screw can effectively reduce the stress of cancellous bone around the screw and enhance the initial stability after fracture operation (Wang et al., 2023). Our data indicate that the fixation strength of screws in the LLP-IRPBC group was significantly higher than that in the LLP-IFS group, with consistent findings in biomechanical experiments. This suggests that the use of bone cement in the LLP-IRPBC group substantially augments the screw’s tensile strength, thereby improving the stability of the fixed structure. This effect is particularly beneficial for older individuals with compromised bone quality, where the connection between screws and Ost bone may exhibit weaker holding force.

The results of biomechanical experiments were compared with those obtained from finite element analysis, these deviations were within a 20% margin. This variance primarily stemmed from the different sources of research subjects for each method: the biomechanical model utilized artificial bionic bone, while the finite element model was based on a human humerus CT image, leading to differences in material properties. Additionally, *in vitro* biomechanical experiments are influenced by environmental factors, operational standards, and the accuracy of measuring instruments. Despite these differences, the overall trends of the two studies were consistent, achieving similar conclusions under identical loading conditions. This consistency validates the effectiveness and reliability of both the finite element and biomechanical models, supporting their use in further research.

Our study presents several limitations. Firstly, the model was developed using a 30-year-old female volunteer, and osteoporosis was simulated solely by modifying the tissue’s elastic modulus. Secondly, this study was based on a skeletal system model, without accounting for the influence of muscles and ligaments. Thirdly, our analysis focused solely on two-part fractures of the proximal humerus and did not consider three-part and four-part fractures. Fourthly, synthetic bone was employed instead of cadaveric bone in the biomechanical experiments to reduce inter-specimen variations. Fifthly, our analysis concentrated on early postoperative structural stability and did not address long-term outcomes. Despite these limitations, “Sandwich” fixation represents an innovative procedure with promising biomechanical properties, which could have valuable implications for clinical decision-making.

## 5 Conclusion

Compared with allogeneic or autologous fibular bone marrow grafting by FEA and biomechanical experiments, “Sandwich” fixation offers several advantages: 1) It provides robust biomechanical stability while ensuring sturdy medial support. 2) “Sandwich” fixation significantly enhances screw fixation strength and effectively prevents screw failure. 3) With the growing adoption of centralized procurement for high-value medical consumables in China, plate costs have significantly reduced, lightening the economic burden of “Sandwich” fixation compared to allogeneic or autologous fibular bone marrow grafting. 4) There are no associated risks of infection or disease transmission. 5) The bone graft within the space between the intramedullary steel plate and the medial cortex contributes to the reconstruction of the humeral head’s normal shape after the fracture has healed.

Proximal humerus fracture in the elderly has always been a difficult point in orthopedic trauma treatment, and no expert consensus has been reached at present. This new approach offers a promising strategy for clinical treatment, and we are eager to build on our findings with further research aimed at solving this problem.

## Data Availability

The original contributions presented in the study are included in the article/Supplementary Material, further inquiries can be directed to the corresponding author.

## Abbreviations

FEA, Finite element analysis; HU, Hounsfield Unit; IFS, Intramedullary fibula segment; IRPBC, Intramedullary reconstruction plate with bone cement; LLP, Lateral locking plate; PHF, Proximal humerus fracture.

## References

[B1] Anglin C. Tolhurst P. Wyss U. P. Pichora D. R. (1999). Glenoid cancellous bone strength and modulus. J. biomechanics 32 (10), 1091–1097. 10.1016/s0021-9290(99)00087-1 10476847

[B2] Badman B. L. Mighell M. (2008). Fixed-angle locked plating of two-three-and four-part proximal humerus fractures. J. Am. Acad. Orthop. Surg. 16 (5), 294–302. 10.5435/00124635-200805000-00008 18460690

[B3] Bergdahl C. Ekholm C. Wennergren D. Nilsson F. Möller M. (2016). Epidemiology and patho-anatomical pattern of 2,011 humeral fractures: data from the Swedish Fracture Register. BMC Musculoskelet. Disord. 17, 159. 10.1186/s12891-016-1009-8 27072511 PMC4830043

[B4] Bergmann G. Graichen F. Bender A. Rohlmann A. Halder A. Beier A. (2011). *In vivo* gleno-humeral joint loads during forward flexion and abduction. J. biomechanics 44 (8), 1543–1552. 10.1016/j.jbiomech.2011.02.142 21481879

[B5] Biermann N. Prall W. C. Böcker W. Mayr H. O. Haasters F. (2019). Augmentation of plate osteosynthesis for proximal humeral fractures: a systematic review of current biomechanical and clinical studies. Archives Orthop. trauma Surg. 139 (8), 1075–1099. 10.1007/s00402-019-03162-2 30903343

[B6] Boesmueller S. Wech M. Gregori M. Domaszewski F. Bukaty A. Fialka C. (2016). Risk factors for humeral head necrosis and non-union after plating in proximal humeral fractures. Injury 47 (2), 350–355. 10.1016/j.injury.2015.10.001 26706457

[B7] Brown C. J. Sinclair R. A. Day A. Hess B. Procter P. (2013). An approximate model for cancellous bone screw fixation. Comput. methods biomechanics Biomed. Eng. 16 (4), 443–450. 10.1080/10255842.2011.624516 22149043

[B8] Chang H. H. Lim J. R. Lee K. H. An H. Yoon T. H. Chun Y. M. (2023). The biomechanical effect of fibular strut grafts on humeral surgical neck fractures with lateral wall comminution. Sci. Rep. 13 (1), 3744. 10.1038/s41598-023-30935-y 36879028 PMC9988971

[B9] Chen H. Ji X. Zhang Q. Liang X. Tang P. (2015). Clinical outcomes of allograft with locking compression plates for elderly four-part proximal humerus fractures. J. Orthop. Surg. Res. 10, 114. 10.1186/s13018-015-0258-9 26195025 PMC4509847

[B10] Chen H. Yin P. Wang S. Li J. Zhang L. Khan K. (2018). The augment of the stability in locking compression plate with intramedullary fibular allograft for proximal humerus fractures in elderly People. BioMed Res. Int. 2018, 1–8. 10.1155/2018/3130625 PMC6165610 30306087

[B11] Chen H. Zhu Z. G. Li J. T. Chang Z. H. Tang P. F. (2020). Finite element analysis of an intramedulary anatomical strut for proximal humeral fractures with disrupted medial column instability: a cohort study. Int. J. Surg. Lond. Engl. 73, 50–56. 10.1016/j.ijsu.2019.11.026 31783165

[B12] Cui X. Chen H. Ma B. Fan W. Li H. (2019). Fibular strut allograft influences reduction and outcomes after locking plate fixation of comminuted proximal humeral fractures in elderly patients: a retrospective study. BMC Musculoskelet. Disord. 20 (1), 511. 10.1186/s12891-019-2907-3 31679513 PMC6825724

[B13] Gardner M. J. Boraiah S. Helfet D. L. Lorich D. G. (2008). Indirect medial reduction and strut support of proximal humerus fractures using an endosteal implant. J. Orthop. trauma 22 (3), 195–200. 10.1097/BOT.0b013e31815b3922 18317054

[B14] Hart N. H. Nimphius S. Rantalainen T. Ireland A. Siafarikas A. Newton R. U. (2017). Mechanical basis of bone strength: influence of bone material, bone structure and muscle action. J. Musculoskelet. neuronal Interact. 17 (3), 114–139. 28860414 PMC5601257

[B15] He Y. He J. Wang F. Zhou D. Wang Y. Wang B. (2015). Application of additional medial plate in treatment of proximal humeral fractures with unstable medial column: a finite element study and clinical practice. Medicine 94 (41), e1775. 10.1097/MD.0000000000001775 26469918 PMC4616805

[B16] He Y. Zhang Y. Wang Y. Zhou D. Wang F. (2017). Biomechanical evaluation of a novel dualplate fixation method for proximal humeral fractures without medial support. J. Orthop. Surg. Res. 12 (1), 72. 10.1186/s13018-017-0573-4 28499398 PMC5429529

[B17] Jabran A. Peach C. Ren L. (2018). Biomechanical analysis of plate systems for proximal humerus fractures: a systematic literature review. Biomed. Eng. online 17 (1), 47. 10.1186/s12938-018-0479-3 29703261 PMC5923007

[B18] Kayabasi O. Ekici B. (2007). The effects of static, dynamic and fatigue behavior on three-dimensional shape optimization of hip prosthesis by finite element method. Mater. Des. 28 (8), 2269–2277. 10.1016/j.matdes.2006.08.012

[B19] Khellafi H. Bouziane M. M. Djebli A. Mankour A. Bendouba M. Bachir Bouiadjra B. A. (2019). Investigation of mechanical behaviour of the bone cement (PMMA) under combined shear and compression loading. J. Biomimetics, Biomaterials Biomed. Eng. 41, 37–48. 10.4028/www.scientific.net/jbbbe.41.37

[B20] Kim Y. H. Kim J. S. Cho S. H. (2001). Strain distribution in the proximal human femur. J. bone Jt. Surg. Br. 83 (2), 295–301. 10.1302/0301-620x.83b2.10108 11284584

[B21] Larsson S. Stadelmann V. A. Arnoldi J. Behrens M. Hess B. Procter P. (2012). Injectable calcium phosphate cement for augmentation around cancellous bone screws. *in vivo* biomechanical studies. J. biomechanics 45 (7), 1156–1160. 10.1016/j.jbiomech.2012.02.004 22386107

[B22] Laux C. J. Grubhofer F. Werner C. M. L. Simmen H. P. Osterhoff G. (2017). Current concepts in locking plate fixation of proximal humerus fractures. J. Orthop. Surg. Res. 12 (1), 137. 10.1186/s13018-017-0639-3 28946902 PMC5613450

[B23] Lee S. H. Han S. S. Yoo B. M. Kim J. W. (2019). Outcomes of locking plate fixation with fibular allograft augmentation for proximal humeral fractures in osteoporotic patients: comparison with locking plate fixation alone. bone and Jt. J. 101-B (3), 260–265. 10.1302/0301-620X.101B3.BJJ-2018-0802.R1 30813788

[B24] McCalden R. W. McGeough J. A. Barker M. B. Court-Brown C. M. (1993). Age-related changes in the tensile properties of cortical bone. The relative importance of changes in porosity, mineralization, and microstructure. J. bone Jt. Surg. Am. 75 (8), 1193–1205. 10.2106/00004623-199308000-00009 8354678

[B25] Mease S. J. Kraeutler M. J. Gonzales-Luna D. C. Gregory J. M. Gardner M. J. Choo A. M. (2021). Current controversies in the treatment of geriatric proximal humeral fractures. J. bone Jt. Surg. Am. 103 (9), 829–836. 10.2106/JBJS.20.00665 33617160

[B26] Panchal K. Jeong J. J. Park S. E. Kim W. Y. Min H. K. Kim J. Y. (2016). Clinical and radiological outcomes of unstable proximal humeral fractures treated with a locking plate and fibular strut allograft. Int. Orthop. 40 (3), 569–577. 10.1007/s00264-015-2950-0 26257277

[B27] Robinson C. M. Page R. S. Hill R. M. Sanders D. L. Court-Brown C. M. Wakefield A. E. (2003). Primary hemiarthroplasty for treatment of proximal humeral fractures. J. bone Jt. Surg. Am. 85 (7), 1215–1223. 10.2106/00004623-200307000-00006 12851345

[B28] Sproul R. C. Iyengar J. J. Devcic Z. Feeley B. T. (2011). A systematic review of locking plate fixation of proximal humerus fractures. Injury 42 (4), 408–413. 10.1016/j.injury.2010.11.058 21176833

[B29] Südkamp N. Bayer J. Hepp P. Voigt C. Oestern H. Kääb M. (2009). Open reduction and internal fixation of proximal humeral fractures with use of the locking proximal humerus plate. Results of a prospective, multicenter, observational study. J. bone Jt. Surg. Am. volume 91 (6), 1320–1328. 10.2106/JBJS.H.00006 19487508

[B30] Sun Q. Wu X. Wang L. Cai M. (2020). The plate fixation strategy of complex proximal humeral fractures. Int. Orthop. 44 (9), 1785–1795. 10.1007/s00264-020-04544-7 32535700

[B31] Wang H. Rui B. Lu S. Luo C. Chen Y. Chai Y. (2019). Locking Plate use with or without strut support for varus displaced proximal humeral fractures in elderly patients. JB JS open access 4 (3), e0060–e0068. 10.2106/JBJS.OA.18.00060 PMC6766384 31592502

[B32] Wang W. B. Yuan X. H. Fu Q. S. Han X. Y. (2023). Zhongguo gu shang = China J. Orthop. traumatology 36 (3), 262–267. 10.12200/j.issn.1003-0034.2023.03.013 36946020

[B33] Zettl R. Müller T. Topp T. Lewan U. Krüger A. Kühne C. (2011). Monoaxial versus polyaxial locking systems: a biomechanical analysis of different locking systems for the fixation of proximal humeral fractures. Int. Orthop. 35 (8), 1245–1250. 10.1007/s00264-011-1220-z 21301828 PMC3167442

[B34] Zheng C. Ma H. Y. Du Y. Q. Sun J. Y. Luo J. W. Qu D. B. (2020). Finite element assessment of the screw and cement technique in total knee arthroplasty. BioMed Res. Int. 2020, 1–7. 10.1155/2020/3718705 PMC7584958 33123571

[B35] Zhu L. Liu Y. Yang Z. Li H. Wang J. Zhao C. (2014). Locking plate fixation combined with iliac crest bone autologous graft for proximal humerus comminuted fracture. Chin. Med. J. 127 (9), 1672–1676. 10.3760/cma.j.issn.0366-6999.20133104 24791873

[B36] Zhu Z. Chang Z. Zhang W. Nie S. Qi L. Tang P. (2023a). How to improve the biomechanical stability of endosteal augmentation for proximal humerus fracture with osteopenia? A cadaveric study. Clin. Biomech. (Bristol, Avon) 101, 105850. 10.1016/j.clinbiomech.2022.105850 36493692

[B37] Zhu Z. Chang Z. Zhang W. Qi H. Guo H. Li J. (2023b). Biomechanical evaluation of novel intra- and extramedullary assembly fixation for proximal humerus fractures in the elderly. Front. Bioeng. Biotechnol. 11, 1182422. 10.3389/fbioe.2023.1182422 37936824 PMC10627012

